# Inter-Group Conflict and Cooperation: Field Experiments Before, During and After Sectarian Riots in Northern Ireland

**DOI:** 10.3389/fpsyg.2015.01790

**Published:** 2015-11-27

**Authors:** Antonio S. Silva, Ruth Mace

**Affiliations:** Department of Anthropology, University College LondonLondon, UK

**Keywords:** parochial altruism, real world behavior, donations, in-group favoritism, cooperation and conflict, evolution of cooperation

## Abstract

The idea that cooperative groups out-compete less cooperative groups has been proposed as a theoretical possibility for the evolution of cooperation through cultural group selection. Previous studies have found an association between increased cooperation and exposure to inter-group violence, but most have not been able to identify the specific target of cooperation and are based on correlational data making it difficult to establish causality. In this study we test the hypothesis that inter-group conflict promotes parochial altruism (i.e., in-group altruism and out-group hostility) by using longitudinal data of a real-world measure of cooperation—charity and school donations—sampled before, during and after violent sectarian riots between Catholics and Protestants in Belfast, Northern Ireland. We find that conflict is associated with reductions in all types of cooperation, with reduced donations to a neutral charity, and both in-group and out-group primary schools. After the conflict, both in-group and out-group donations increased again. In this context we find no evidence that inter-group conflict promotes parochial altruism.

## Introduction

Inter-group competition is often put forward as a prominent factor in the evolution of cooperation (Boorman and Levitt, 1973; Choi and Bowles, 2007; Bowles, 2009). Specifically, models of cultural group selection depend on competition between groups for traits that favor the group to evolve, in which groups compete over access to resources such as food, mates or territory (Bowles et al., 2003; Choi and Bowles, 2007; García and van den Bergh, 2011). Cultural group traits that provide an advantage to groups in conflict, such as altruism, will proliferate at the expense of other cultural traits that do not, eventually leading to group extinction through conquest and assimilation (Henrich, 2004). In these theoretical models of the evolution of cooperation through inter-group conflict, biased altruism toward the in-group co-evolves alongside out-group hostility—in what is termed parochial altruism—as a way of groups maximizing their payoffs (Bowles et al., 2003; Choi and Bowles, 2007; García and van den Bergh, 2011). In these models, inter-group conflict promotes the co-evolution of in-group altruism and out-group hostility, which leads to the logical inference that in situations of conflict levels of in-group altruism should be negatively associated with levels of out-group altruism (Arrow, 2007; Choi and Bowles, 2007).

The findings from the models pointing to an association between parochial altruism and inter-group conflict are also supported by empirical data in both the lab and field. Several studies have shown increased in-group altruism and social cohesion in response to violent conflict, in which individuals who had experienced violence were found to be more cooperative in experimental scenarios than individuals without exposure to violence (Bellows and Miguel, 2009; Gilligan et al., 2011; Gneezy and Fessler, 2011; Voors et al., 2012; Bauer et al., 2014). While it should be noted that this type of cooperative behavior is not necessarily associated with altruism *sensu stricto* (i.e., lifetime fitness costs to the actor) as described in the models of parochial altruism (Bowles et al., 2003; Choi and Bowles, 2007; García and van den Bergh, 2011), the findings from these studies are normally put forward as supporting empirical evidence for the theoretical models of parochial altruism (Bernhard et al., 2006; Puurtinen and Mappes, 2009; Gneezy and Fessler, 2011; Voors et al., 2012; Bauer et al., 2014). In contrast to these findings, our previous study using naturalistic measures of cooperation in Northern Ireland found that exposure to inter-group conflict between Catholics and Protestants was associated with reduced donations to out-group schools and the return of out-group lost letters, but there was no evidence that it influenced in-group cooperation. Rather, socio-economic status was the major determinant of cooperative behavior (Silva and Mace, 2014).

One possibility for the conflicting results is that studies finding increased levels of cooperation associated with inter-group conflict are based on economic games and do not use real life groups with a history of conflict in their experimental set-up. Instead they employ abstract concepts of in-group and out-group, such as children from the same classroom as in-group and children from a different school as out-group (Bauer et al., 2014) or anonymous neighbors who may or may not have shared group membership (Voors et al., 2012). Furthermore, these studies do not use a control group and are not able to distinguish between different types of cooperative behavior by conflating in-group cooperative behavior with unbiased cooperation and—with the exception of Bauer et al. (2014)—also fail to measure out-group altruism. The accurate identification of the specific type of cooperation is crucial, as the hypotheses for the evolution of cooperation through inter-group conflict require cooperation to be biased toward the in-group, not to be indiscriminately applied (Arrow, 2007; Bauer et al., 2014).

Our previous study in Belfast addressed some of these issues by determining the role of conflict on cooperation toward in-group, out-group, and neutral institutions using naturalistic measures of cooperation, donations and lost letters (Silva and Mace, 2014). However, Silva and Mace (2014) was still reliant on cross-sectional data and therefore limited in being able to establish a causal link between inter-group conflict and parochial altruism. To our knowledge, Gneezy and Fessler (2011) is the only study that has looked into this relationship using longitudinal data. They conducted ultimatum (UGs) and trust games (TGs) between Israeli senior citizens before, during and after the 2006 Israel–Hezbollah war and found that during the war participants were more likely to reject low offers in the UGs, and transfer back more money if the initial offer was high in TGs. There were no significant differences for the initial amounts offered in either game. These results were interpreted as evidence that in wartime people are more likely to incur a cost to reward cooperative behavior and punish within-group uncooperative behavior.

The study in Israel provides an interesting, if partial, insight into how cooperation is affected by inter-group conflict. First, the lack of significant differences in the initial amounts offered over time suggest that cooperative tendencies may have remained unchanged through the conflict; although the interpretation of these behaviors is complicated as selfish strategic considerations in UGs and TGs can also result in increased offers (Dawes et al., 2007; Brañas-Garza et al., 2014). Second, the games were conducted at the same time as the Lebanon and Israel war, but only between Israeli senior citizens of the same ethnic group living in a housing facility in Tel Aviv. No salient group affiliation was used, so it is not possible to establish how conflict affects cooperation differently toward the in-group or out-group.

In this study, we use a naturalistic donation experiment to assess how a temporary sharp increase in violence between Catholics and Protestants in Northern Ireland affects cooperation toward the in-group, out-group and an unbiased institution, which is used as a control group. The context of Northern Ireland provides a valuable case study on the dynamics of inter-group interactions. These two groups have an on-going history of violence that has resulted in over 3500 people being killed (Sutton, 2012) and tens of thousands injured (Breen-Smyth, 2012) in the past decades, alongside marked levels of residential and education segregation, with the majority of the population today living in areas made up of over 80% of their own religious group (Byrne et al., 2006) and 94% of all children attending predominantly Catholic (run by the Catholic Church) or Protestant (run by the state or Protestant Churches) schools. The use of donations to primary schools in the experiments intends to reflect actual inter-group grievances between Catholics and Protestants in Northern Ireland associated with school funding (BBC News, 2001). The individuals in the study are not aware that the donations are part of an experiment, minimizing the artificiality typical of most lab and field based economic games.

In this study, we make use of an eruption of sectarian violence that started in December 2012 over a dispute related to the flying of Union flag in public buildings.

On the 3rd December 2012, the Belfast City Council passed a motion to restrict the flying of the Union flag to 18 designated days in the Belfast City Hall (Belfast City Council, 2012). The flag had previously been flown all year round and this change sparked protests from the Protestant community (who mostly feels an affinity with the United Kingdom), leading to an escalation of violence throughout the region, which resulted in violent riots over the next few months. The riots spread through the city with buses being set alight, cars being hijacked and skirmishes between Protestants, Catholics and the police involving water cannons, rocks, and petrol bombs (BBC News, 2013b). During this period, numerous violent clashes led to 560 people being charged and arrested (BBC News, 2013a), 157 police men and women injured and an estimated £70 million costs in material damages, reduced business revenues and increased policing (BBC News, 2013c, 2014).

The violent clashes in Belfast continued through January and at this time we went back to Belfast to repeat the survey and donations experiment previously conducted in May 2012 during a more peaceful time. We then went back again in May and June 2013 to investigate the aftermath of the riots. This allowed us to have a longitudinal dataset of cooperative behavior and attitudes at the neighborhood level that now enables us to assess the role of inter-group conflict on cooperation in a quasi-experimental framework.

## Materials and methods

**Figure 1:**
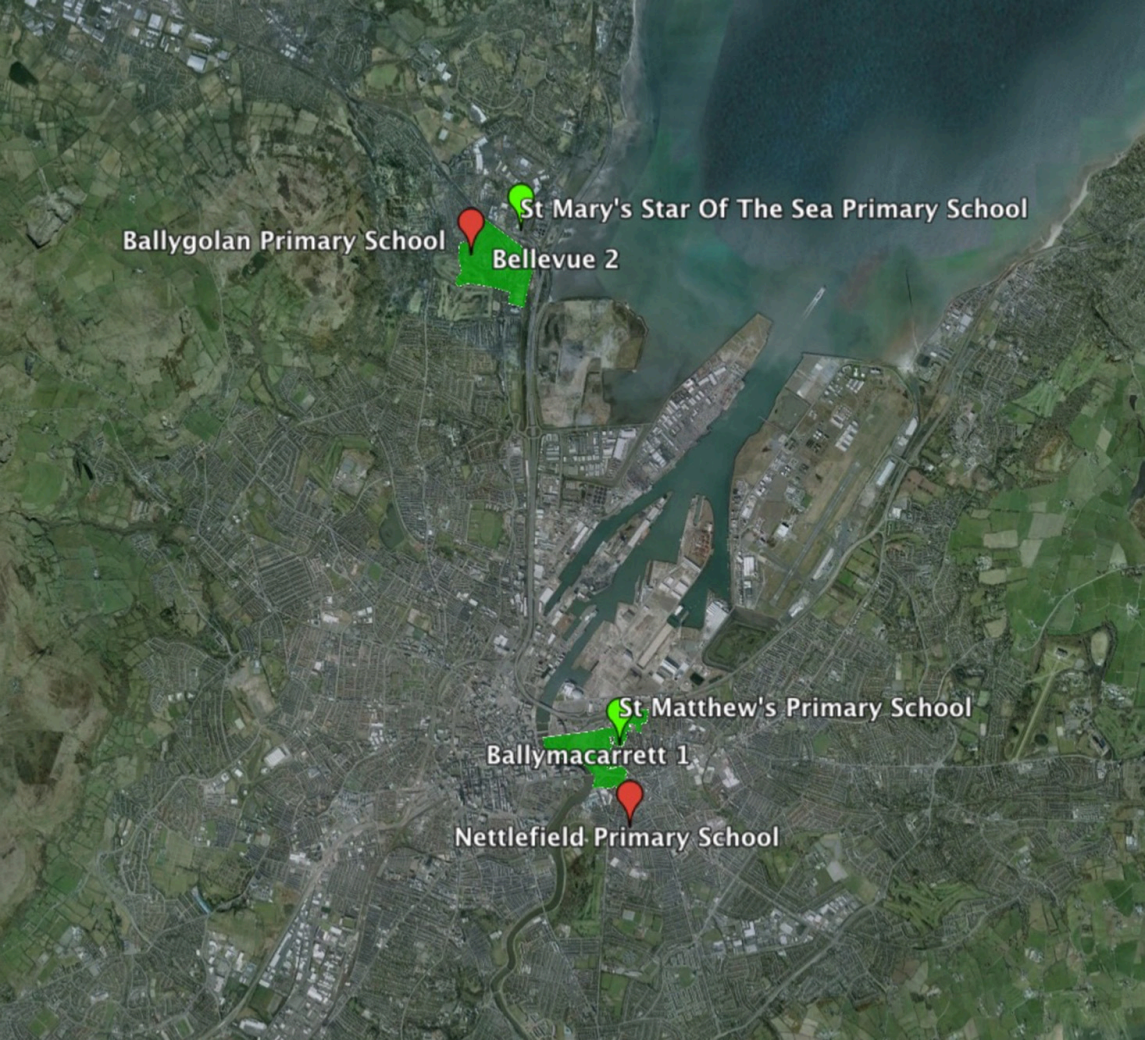
**Map of Belfast with the neighborhoods Ballymacarrett 1 and Bellevue 2 in green and the 4 primary schools used in the donations experiments before, during and after the sectarian riots**. Catholic primary schools (green markers) and Protestant primary schools (red markers).

We conducted an impromptu natural experiment when sectarian riots erupted in Belfast in January 2013 by conducting the survey and donation experiments at the time of the riots in two previously sampled neighborhoods, Ballymacarrett 1 and Bellevue 2. The neighborhoods represent the UK Census lower super output areas, a UK standard geographic unit generated taking into account “population size, mutual proximity and social homogeneity” (ONS, 2005, 2) (Figure 1). We also conducted the surveys and donations experiments in the aftermath of the riots in the same two neighborhoods in May and June 2013. We conducted a total of 228 donations experiments, including 49 donations experiments in the pre-riot period, 77 during the riots and 102 after the riots (4 donations data points were not included in the final analysis due to missing covariate data).

The survey was completed in person by 6 trained assistants at the houses of the respondents between 10.00 and 20.00. Each assistant was allocated a set of streets in the neighborhood and then knocked on doors asking residents if they would like to take part in the survey. The total number of attempts and responses were only recorded during 14 days in May and June 2013 in Ballymacarrett 1 and Bellevue 2 and from this sample out of a total of 1267 attempts, there was no answer on 69% of the houses, 23% refused to take part and 8% filled in the questionnaire, which matched our subjective personal experience from the previous sampling periods (see Table S1 for the sample representativeness).

The questionnaire consisted of 50 questions, required about 10 minutes to complete and was structured with multiple-choice responses that the researcher read out and for which the respondent chose the most appropriate choice. The questionnaire addressed a range of issues with a focus on questions about the respondents' socio-economic characteristics status (S.E.S.) and experiences of the conflict, specifically questions on whether the individual had been attacked or felt threatened by the other group. We used these variables to create a sectarian threat index from a factor analysis of variables related to the individual exposure to sectarian attacks and threat, which we used as a measure of inter-group conflict (see Table S3 for more detail on this factor variable) in addition to the time of the riots.

The donations experiment was conducted immediately after the completion of the questionnaire. The participants were informed in the beginning that they would receive a £5 financial incentive at the end for completing the questionnaire and could choose to donate or keep the money. After the completion of the questionnaire, the researcher handed the participant the financial incentive in the form of 5 pound coins and presented in view of the participant a charity box with the name of the local school or charity (Figure S1), where the participant can drop some or all of the coins (see SI for protocol). There were three treatments—one for each of the local schools and one for the charity—and participants were only given the choice to donate to one of the three options, which was randomly allocated, making it a between-subject experimental design. The amount donated to the local school treatments measures in-group (if participant is of the same religion as the school) and out-group cooperation (if participant is of a different religion as the school), and the charity treatment measures unbiased cooperation (see Table S2 for raw data). The school donation is a natural experiment that has essentially the same payoff structure as a dictator game (Kahneman et al., 1986), albeit one that is administered surreptitiously and involves real life cooperative behavior involving donating to an institution rather than an individual. The selection of the primary schools was conducted by choosing the nearest Catholic and Protestant school to the centroid of the neighborhood using Google Maps. This study was approved by the UCL Research Ethics Committee (ID: 2390/002) and all participants provided written informed consent to take part in the research.

## Analysis

The main hypothesis is derived from the theoretical models of inter-group conflict and parochial altruism (Bowles et al., 2003; Choi and Bowles, 2007; García and van den Bergh, 2011), which predict that conflict promotes increased in-group cooperation and reduced out-group cooperation (i.e. parochial altruism). Specifically, we predict that parochial altruism will increase during the riots in comparison to before and after the riots.

We ran four linear regressions to predict the (i) overall and the specific amount donated over time to (ii) the unbiased charity (*Save the Children*), (iii) the in-group, and (iv) the out-group primary schools. The continuous outcome variable was the amount donated in British pounds. The main explanatory variable was the time of the sampling (dummy coded as pre-riot, mid-riot and post-riot). We also control for household income, highest educational level achieved, age, gender, religious background, and neighborhood. We also ran a model with the individual sectarian threat as an alternative explanatory variable to determine how sectarian threat affects donations overall.

We performed a manipulation check to determine if the riots caused a different shift in people's perception of sectarian threat using a linear regression with the factor sectarian threat as the outcome variable.

## Results

**Figure 2:**
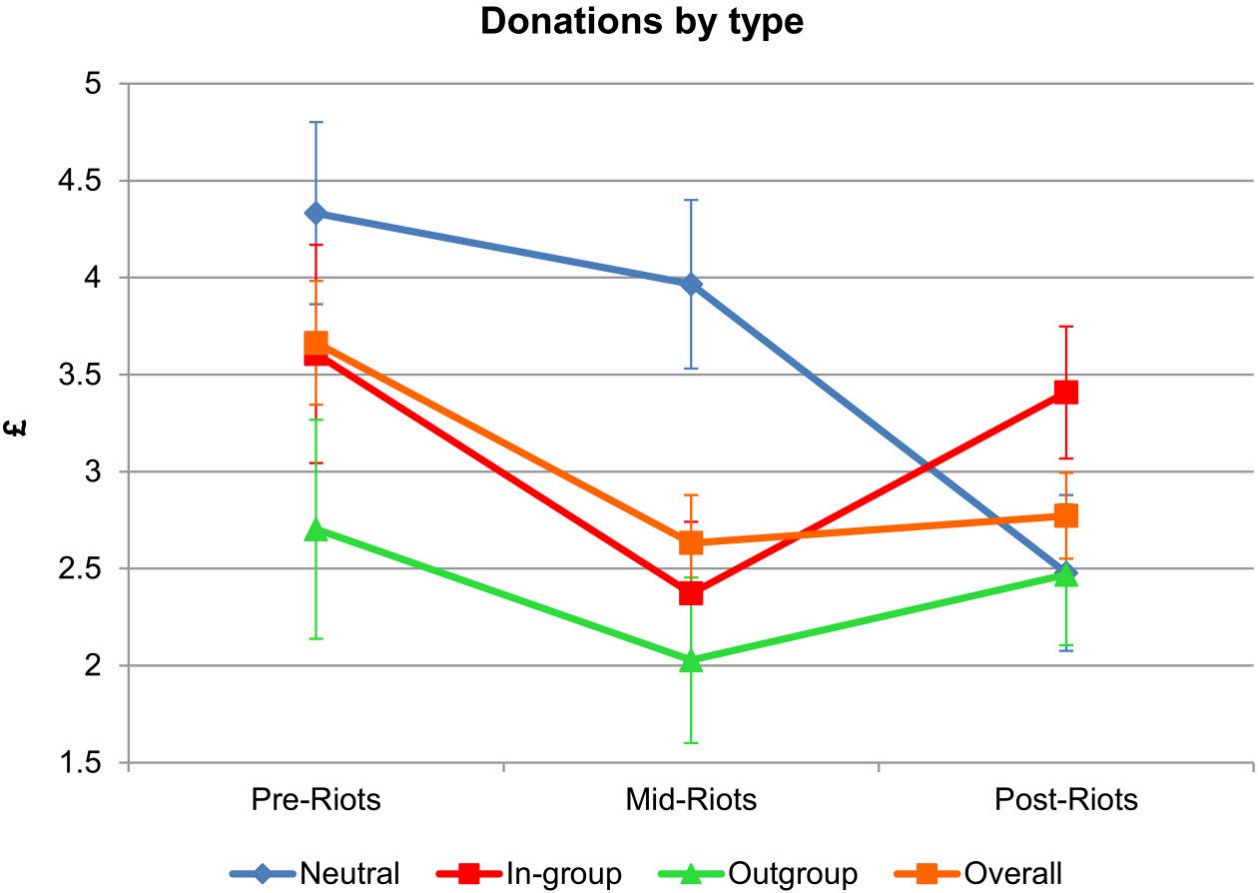
**Predicted donations by type over time**. Predicted value (£) of an individual donating to the neutral charity *Save the Children*, an in-group primary school, an out-group primary school and all combined donations over time (before, during, and after the riots). These predicted values are controlled for individual household income, educational level, age, gender and religion. Error bars represent the standard errors.

There was a significant reduction in overall donations during the riots compared to before the riots (β = −1.03 [−1.83; −0.24], *p* < 0.05; Table S4 and Figure 2). When looking at the different types of donations, we find that in-group donations suffered the most during the riots, with £1.23 less being given to in-group primary schools during the riots compared to before the riots (β = −1.23 [−2.58; 0.11], *p* < 0.1; Table S4 and Figure 2). After the riots, donations to the in-group and to the out-group increased again (β = 1.04 [0.02; 2.06], *p* < 0.05; β = 0.44 [−0.69; 1.58], *p* > 0.1), but only the increase of in-group donations was significant at conventional levels (Table S4 and Figure 2). Household income significantly predicted increased levels of cooperation, with high-income individuals donating 84 p more than low-income individuals (β = 0.84 [0.09; 1.59], *p* < 0.05; Table S4 and Figure 2)

People experienced a marginally significant increase in the feelings of sectarian threat during the riots, compared to before and after the riots (β = 0.25 [−0.03, 0.52], *p* < 0.1; Table S5). Sectarian threat is felt most by young people (β = −0.01 [−0.02, −0.00], *p* < 0.01; Table S5) and Protestants feel marginally less threatened than Catholics (β = −0.18 [−0.38, 0.03], *p* < 0.01; Table S5). Overall, individuals with higher levels of sectarian threat were significantly less likely to donate to out-group primary schools (β = −0.97 [−1.73, −0.20], *p* < 0.05), but sectarian threat had no impact on in-group or neutral donations (Table S6).

## Discussion

Overall, there was a significant trend toward a reduction in cooperative behavior during the riots, which suggests that in the context of Northern Ireland inter-group conflict does not promote cooperation. There is a marked decline in all types of cooperation in both neighborhoods during increased inter-group conflict. Specifically, the decline in in-group cooperation is most substantial during the riots with an average of £1.23 less donations to an in-group primary school than before the riots. The overall levels of cooperation remained lower in the aftermath of the riots compared to before, but the levels of in-group and out-group donations appear to be returning back toward the original levels. This suggests that the impact of this conflict may not be long lasting and cooperation can return to normal levels after a few months. This may be especially true in the context of Northern Ireland where people might be somewhat desensitized to sectarian violence with inter-group tension always present and low-level conflict between the two groups being a frequent occurrence.

In contrast with previous studies, the results from this study do not support the hypothesis that conflict promotes cooperation. In relation to Gneezy's and Fessler's (2011) results, the differences may be related to the fact that they use ultimatum and trust games to measure punishment and trusting behavior, while this study focuses on cooperative behavior. The behavior in ultimatum and trust games is difficult to interpret as it can stem from various psychological mechanisms other than altruistic preferences, such as status seeking, spite, or fairness (Dawes et al., 2007; Brañas-Garza et al., 2014). The concepts of cooperation and punishment are often assumed to be linked (Boyd et al., 2003; Bernhard et al., 2006; Hauert et al., 2007), but recent evidence points to a lack of association between propensity of cooperation and punishment within individuals (Yamagishi et al., 2012; Brañas-Garza et al., 2014; Peysakhovich et al., 2014). It is possible that conflict increases the propensity to punish, although it is not clear whether this would be directed toward the in-group or the out-group (Bernhard et al., 2006; Mathew and Boyd, 2011) and no out-group members were included in the Israel study.

Our study is the first to test the longitudinal effect of conflict on cooperation using real-world measures and groups, so it is also possible that previous results are artifacts from the use of cross-sectional economic games. The validity of traditional economic games as measures of human cooperative behavior has started to be questioned with multiple studies failing to find correlations between behavior in experimental games and in real life measures in the field (Laury and Taylor, 2006; Levitt and List, 2007; Benz and Meier, 2008). These games may cue the subjects to play according to specific real life cooperative social norms that are not particularly relevant to the hypothesis being tested (Laury and Taylor, 2006; Binmore, 2010). Furthermore, participants in games may not understand the cost and benefits inherent to the games' processes making it difficult to interpret their behavior (Burton-Chellew and West, 2013). This study highlights the importance of capturing real life cooperative behavior using natural experiments in the field to understand the effects of inter-group conflict on cooperation.

Current theoretical models of parochial altruism build on the assumption that increased pro-sociality or in-group altruism results in a group advantage in a situation of inter-group conflict by setting the cost accrued by the in-group altruist to always be lower than the benefit accrued to the group (or another individual in the group) (Bowles et al., 2003; Choi and Bowles, 2007; García and van den Bergh, 2011). Lab based empirical results supporting these models are also based on a game payoff structure in which altruistic groups always out-compete selfish groups in a situation of group conflict (Bornstein, 2003; Puurtinen and Mappes, 2009). Here, we question whether these assumptions are realistic and argue that it is not generalizable to all situations where groups are in competition or conflict. In the case of Catholics and Protestants in Northern Ireland, recent conflict between the two groups has mostly been over issues related to schools, housing and symbolic displays (Nolan, 2012); it is possible that in these situations increased group cohesion does not provide a group advantage, or that the individual cost of helping the group out-weighs the potential group advantage. Conflict appears to have a negative impact on cooperation, arguably in a similar way to other adverse environments that affect levels of inter-personal trust—such as income deprivation and low levels of social capital—and that also lead to a reduction in cooperative behavior (Putnam, 2000; Holland et al., 2012).

The results from this study show that the effects of conflict may be multi-faceted. The levels of sectarian threat as measured by the survey questions appear to mostly affect cooperation toward the out-group, corroborating the results from the cross-sectional data in Silva and Mace (2014). However, the effects of conflict may not be entirely captured by these survey measures as the riots lead to a reduction of all types of cooperation and not just toward the out-group. It is also important to note that the sample sizes used are small (although comparable to Gneezy and Fessler, 2011) and as result the findings from this study alone are not conclusive. These results do, however, strengthen the findings from the cross-sectional data (Silva and Mace, 2014) and together do not support the models of inter-group conflict and parochial altruism, putting into question the theoretical idea that cooperation could have evolved through increased group pay-offs via inter-group conflict (Bowles et al., 2003; Choi and Bowles, 2007; García and van den Bergh, 2011).

In situations of conflict, individuals may not necessarily behave altruistically and there are perhaps more evolutionarily parsimonious explanations for the behavior of individuals during conflict, such as reputation concerns (Nowak and Sigmund, 1998), enforcement mechanisms (Mathew and Boyd, 2011) or hierarchical dominance structures (Guala, 2012). It may also be that only behaviors directly related to the threat in question (such as joining the army in the face of military invasion) are influenced by external threat, rather than more generalized cooperation. A recent review of inter-group warfare in small scale societies found that individual benefits—mostly related to reputation and status—better explain the intensity of conflict than group-level benefits (Glowacki and Wrangham, 2013). Our findings also suggest that altruism may not be an important motivation in inter-group conflict and demonstrate how conflict can have a pernicious effect on all types of cooperative behavior.

### Conflict of interest statement

The authors declare that the research was conducted in the absence of any commercial or financial relationships that could be construed as a potential conflict of interest.
