# Supplementary material for: Inter-Group Conflict and Cooperation: Field Experiments Before, During and After Sectarian Riots in Northern Ireland
Source: Front Psychol. 2015 Nov 27;6:1790. doi: 10.3389/fpsyg.2015.01790 (PMC4661283; doi:10.3389/fpsyg.2015.01790)
Supplement: Supplementary file 1 [file Table1.PDF]

Table S1. Sample characteristics over time. Percentage distribution of household income, highest educational level achieved, employment status, gender, religious background and mean age of the individuals in samples over time and the Census 2011 data for the same neighbourhoods. * - the unemployed data for the Census corresponds to unemployed individuals of active age, disabled or sick and their full-time carers

Variable	Census 2011	Overall	Pre-Riot	Mid-Riot	Post-Riot
	%	% (*n*)	% (*n*)	% (*n*)	% (*n*)
**Low HH Income**	-	43 (98)	57.1 (28)	45.5 (35)	34.3 (35)
**Mid HH Income**	-	24.6 (56)	22.5 (11)	23.4 (18)	26.5 (27)
**High HH Income**	-	32.5 (74)	20.4 (10)	31.2 (24)	39.2 (40)
**Primary School**	40.6	20.9 (47)	40.8 (20)	24.7 (19)	8.1 (8)
**GCSE**	31.1	46.75 (105)	40.8 (20)	44.2 (34)	51.5 (51)
**A-Level**	12.9	21.3 (48)	12.2 (6)	23.4 (18)	24.2 (24)
**Undergraduate**	16.25	11.1 (25)	6.1 (3)	7.8 (6)	16.2 (16)
**Unemployment**	33.2 *	43.4 (99)	44.9 (22)	48.1 (37)	39.2 (40)
**Female**	51.9	55.1 (125)	61.2 (30)	52.6 (40)	53.9 (55)
**Male**	47.1	44.9 (102)	38.8 (19)	47.4 (36)	46.1 (47)
**Catholic**	61.3	55.7 (127)	46.9 (23)	52.0 (40)	62.8 (64)
**Protestant**	31.2	44.3 (101)	53.1 (26)	48.0 (37)	37.3 (38)
		$\bar{x}$ (δ)	$\bar{x}$ (δ)	$\bar{x}$ (δ)	$\bar{x}$ (δ)
**Age**	-	43.8 (17.3)	42.0 (14.9)	47.5 (18.9)	42.0 (16.9)
**Observations**		228	49	77	102
