# Supplementary material for: Inter-Group Conflict and Cooperation: Field Experiments Before, During and After Sectarian Riots in Northern Ireland
Source: Front Psychol. 2015 Nov 27;6:1790. doi: 10.3389/fpsyg.2015.01790 (PMC4661283; doi:10.3389/fpsyg.2015.01790)
Supplement: Supplementary file 2 [file Table2.PDF]

Table S2. Frequency of donations over time. Frequency of donations by amount in pound sterling to the neutral charity (Save the Children), in-group, out-group primary school before, during and after the sectarian riots.

Donations	Pre-Riot	Mid-Riot	Post-Riot
**Neutral (£)**			
0	2	2	4
1	0	3	5
2	0	0	6
3	0	0	1
4	0	0	0
5	15	14	9
**In-group (£)**			
0	5	8	3
1	0	6	4
2	0	3	6
3	0	1	3
4	0	1	0
5	10	11	22
**Out-group (£)**			
0	7	10	12
1	0	4	3
2	1	4	9
3	0	2	1
4	0	0	0
5	9	8	14
**Observations**	49	77	102
