# Supplementary material for: Inter-Group Conflict and Cooperation: Field Experiments Before, During and After Sectarian Riots in Northern Ireland
Source: Front Psychol. 2015 Nov 27;6:1790. doi: 10.3389/fpsyg.2015.01790 (PMC4661283; doi:10.3389/fpsyg.2015.01790)
Supplement: Supplementary file 3 [file Table3.PDF]

Sectarian Threat Index

The level of inter-group threat is measured through a polychoric factor analysis of variables related to whether the individual had been attacked or felt threatened by the other group. The mean value is 1.8 (s.d.=0.9), ranging from 0.7 (low threat) to 4.0 (high threat).

Table S3. List of the component variables of the factor sectarian threat index, associated questionnaire questions and percentage distribution of the responses (value in brackets). It also includes the factor loadings and unique variances of the variables in the factor.

**Sectarian Threat Index (*n*=218)**		
**Uncomfortable in different neighbourhood**	**Factor loading**	**Uniqueness**
Would you feel uncomfortable walking around in certain neighbourhoods because you feel people there are from a different religion to your own?	0.48	0.77
**Response**	**Percentage**	
Yes (2)	55.3	
No (0)	43.9	
Not sure (1)	0.9	
**Community under threat**	**Factor loading**	**Uniqueness**
Do you feel that your community is currently under threat from others outside of it?	0.77	0.40
**Response**	**Percentage**	
Yes (2)	41.0	
No (0)	57.7	
Not sure (1)	1.3	
**Reduced Segregation**	**Factor loading**	**Uniqueness**
Would you agree that the segregation between religious communities is less pronounced since the Good Friday agreement?	0.42	0.82
**Response**	**Percentage**	
Strongly agree (1)	3.1	
Agree (2)	38.7	
Neither agree nor disagree (3)	33.8	
Disagree (4)	17.8	
Strongly disagree (5)	6.7	

Sectarian Attack	Factor loading	Uniqueness
In the past year have you been attacked, threatened or insulted because of your religious/political background?	0.70	0.51
**Response**	**Percentage**	
No (0)	80.2	
Rarely (1)	5.0	
A few times (2)	9.0	
Many times (3)	5.9	

Neighbourhood violence	Factor loading	Uniqueness
Please indicate how much of a concern you feel sectarian violence is in your neighbourhood	0.86	0.25
**Response**	**Percentage**	
Not often a problem (1)	33.9	
Sometimes a problem (2)	35.7	
Often a problem (3)	15.0	
Very often a problem (4)	15.4	

Neighbourhood discrimination	Factor loading	Uniqueness
Please indicate how much of a concern you feel sectarian discrimination is in your neighbourhood	0.82	0.33
**Response**	**Percentage**	
Not often a problem (1)	42.7	
Sometimes a problem (2)	30.0	
Often a problem (3)	16.7	
Very often a problem (4)	10.6	
