# Supplementary material for: Inter-Group Conflict and Cooperation: Field Experiments Before, During and After Sectarian Riots in Northern Ireland
Source: Front Psychol. 2015 Nov 27;6:1790. doi: 10.3389/fpsyg.2015.01790 (PMC4661283; doi:10.3389/fpsyg.2015.01790)
Supplement: Supplementary file 4 [file Table4.PDF]

Table S4. Predicted donations by type over time. Coefficients and 95% confidence intervals from linear regressions used to predict the individual amount given in donations over time (before, during and after the riots) to the neutral charity *Save the Children*, and in-group and out-group primary schools. ****p*<0.001; ***p*<0.01; **p*<.0.05; .*p*<0.1

**Donations**	**Overall**	**Neutral**	**In-group**	**Out-group**
	β [CI]	β [CI]	β [CI]	β [CI]
**Pre-Riots**	1.03*	0.37	1.23 .	0.68
(ref. Mid-Riots)	[0.24,1.83]	[-0.93,1.66]	[-0.11,2.58]	[-0.75,2.10]
**Post-Riots**	0.14	-1.49*	1.04*	0.44
(ref. Mid-Riots)	[-0.52,0.80]	[-2.69,-0.28]	[0.02,2.06]	[-0.69,1.58]
**Mid HH Income**	0.60	0.93	0.96	-0.17
(ref. Low HH income)	[-0.12,1.32]	[-0.31,2.17]	[-0.47,2.39]	[-1.44,1.09]
**High HH Income**	0.84*	0.22	1.30*	0.57
(ref. Low HH income)	[0.09,1.59]	[-1.22,1.66]	[0.12,2.49]	[-0.79,1.92]
**GCSE**	-0.30	0.98	-1.10 .	-0.22
(ref. Primary School)	[-1.10,0.51]	[-0.45,2.41]	[-2.40,0.19]	[-1.85,1.41]
**A-Level**	0.12	-0.13	0.28	0.57
(ref. Primary School)	[-0.88,1.12]	[-1.95,1.69]	[-1.28,1.83]	[-1.42,2.56]
**Undergraduate**	0.01	2.40 .	-0.72	-0.03
(ref. Primary School)	[-1.16,1.19]	[-0.23,5.03]	[-2.39,0.95]	[-2.19,2.13]
**Age**	0.00	0.00	0.01	0.00
	[-0.01,0.02]	[-0.03,0.04]	[-0.02,0.04]	[-0.03,0.04]
**Male**	0.27	-0.12	0.35	0.91 .
(ref. Female)	[-0.31,0.85]	[-1.26,1.02]	[-0.57,1.27]	[-0.12,1.95]
**Protestant**	-0.25	-0.92 .	-0.29	0.35
(ref. Catholic)	[-0.83,0.33]	[-1.96,0.13]	[-1.21,0.63]	[-0.71,1.40]
**Bellevue 2**	-0.30	-0.97 .	-0.84 .	0.49
(ref. Ballymacarrett 1)	[-0.87,0.26]	[-2.01,0.07]	[-1.72,0.04]	[-0.50,1.49]
**Constant**	2.01*	3.88*	1.76	-0.07
	[0.35,3.66]	[0.78,6.98]	[-0.84,4.35]	[-3.22,3.08]
Observations	224	59	81	84
