# Supplementary material for: Inter-Group Conflict and Cooperation: Field Experiments Before, During and After Sectarian Riots in Northern Ireland
Source: Front Psychol. 2015 Nov 27;6:1790. doi: 10.3389/fpsyg.2015.01790 (PMC4661283; doi:10.3389/fpsyg.2015.01790)
Supplement: Supplementary file 5 [file Table5.PDF]

Table S5. Sectarian threat over time. Coefficients and 95% confidence intervals from adjusted linear regressions used to predict the levels of individual sectarian threat over time (before, during and after the riots). ***p<0.001; **p<0.01; *p<.0.05; .p<0.1

**Sectarian Threat**	β [CI]
**Mid-Riots** (ref. Pre-Riots)	0.25 [-0.03,0.52] .
**Post-Riots** (ref. Pre-Riots)	-0.02 [-0.30,0.26]
**Mid HH Income** (ref. Low HH income)	0.09 [-0.16,0.35]
**High HH Income** (ref. Low HH income)	0.07 [-0.19,0.33]
**GCSE** (ref. Primary School)	0.02 [-0.27,0.30]
**A-Level** (ref. Primary School)	-0.16 [-0.51,0.20]
**Undergraduate** (ref. Primary School)	0.18 [-0.24,0.61]
**Age**	-0.01 [-0.02,-0.00] **
**Male** (ref. Female)	-0.08 [-0.29,0.12]
**Protestant** (ref. Catholic)	-0.18 [-0.38,0.03] .
**Bellevue 2** (ref. Ballymacarrett 1)	-0.92 [-1.12,-0.72] ***
**Constant**	3.05 [2.47,3.63] ***
**Observations**	214
