# Supplementary material for: Inter-Group Conflict and Cooperation: Field Experiments Before, During and After Sectarian Riots in Northern Ireland
Source: Front Psychol. 2015 Nov 27;6:1790. doi: 10.3389/fpsyg.2015.01790 (PMC4661283; doi:10.3389/fpsyg.2015.01790)
Supplement: Supplementary file 6 [file Table6.PDF]

Table S6. Donations by type and sectarian threat. Coefficients and 95% confidence intervals from adjusted linear regressions used to predict the individual amount given in donations to the neutral charity Save the Children, in-group and out-group primary schools, by individuals levels of sectarian threat. ****p*<0.001; ***p*<0.01; **p*<.0.05; .*p*<0.1

Donations	Overall	Neutral	In-group	Out-group
	β [CI]	β [CI]	β [CI]	β [CI]
**Sectarian Threat**	-0.47*	0.06	-0.15	-0.97*
	[-0.86,-0.07]	[-0.68,0.80]	[-0.75,0.46]	[-1.73,-0.20]
**Mid HH Income**	0.52	1.22 .	1.03	0.09
(ref. Low HH income)	[-0.21,1.25]	[-0.12,2.57]	[-0.39,2.45]	[-1.16,1.34]
**High HH Income**	0.92*	0.07	1.52*	0.80
(ref. Low HH income)	[0.16,1.68]	[-1.49,1.62]	[0.31,2.74]	[-0.52,2.12]
**GCSE**	-0.68 .	0.73	-1.05 .	-0.81
(ref. Primary School)	[-1.46,0.11]	[-0.90,2.36]	[-2.28,0.19]	[-2.38,0.76]
**A-Level**	-0.54	-1.58	-0.11	-0.11
(ref. Primary School)	[-1.53,0.45]	[-3.65,0.49]	[-1.61,1.39]	[-2.03,1.82]
**Undergraduate**	-0.25	1.74	-0.41	-0.31
(ref. Primary School)	[-1.44,0.94]	[-1.11,4.59]	[-2.20,1.39]	[-2.30,1.69]
**Age**	-0.01	0.00	0.00	-0.02
	[-0.03,0.01]	[-0.04,0.04]	[-0.03,0.04]	[-0.05,0.02]
**Male**	0.20	0.36	0.20	0.84
(ref. Female)	[-0.39,0.79]	[-0.94,1.67]	[-0.80,1.19]	[-0.18,1.86]
**Protestant**	-0.25	-1.02 .	-0.31	0.27
(ref. Catholic)	[-0.85,0.34]	[-2.16,0.12]	[-1.30,0.69]	[-0.76,1.30]
**Bellevue 2**	-0.70*	-1.15 .	-0.84	-0.66
(ref. Ballymacarrett 1)	[-1.38,-0.01]	[-2.46,0.16]	[-1.89,0.22]	[-1.96,0.64]
**Constant**	4.42***	3.23	3.31*	4.02*
	[2.52,6.32]	[-0.81,7.27]	[0.48,6.13]	[0.16,7.89]
Observations	214	56	77	81
