# Supplementary material for: Inter-Group Conflict and Cooperation: Field Experiments Before, During and After Sectarian Riots in Northern Ireland
Source: Front Psychol. 2015 Nov 27;6:1790. doi: 10.3389/fpsyg.2015.01790 (PMC4661283; doi:10.3389/fpsyg.2015.01790)
Supplement: Supplementary file 7 [file Presentation1.PDF]

Supplementary Information

Inter-group conflict and cooperation: field experiments before, during and after sectarian riots in Northern Ireland

Antonio S. Silva *, Department of Anthropology, University College London, London, UK

Ruth Mace, Department of Anthropology, University College London, London, UK

*** Correspondence:**
Antonio S. Silva
University College London
Department of Anthropology
14 Taviton Street
WC1H 0BW London
UK

Variables

Education: Ordinal variable on the highest educational level achieved

Gender: Nominal variable of gender

Household income: Ordinal variable of the terciles of household income in pounds equivalised using the OECD modified scale to adjust for household size and composition (Hagenaars et al. 1996)

Religion: Binary variable of the religious background in which the individual was brought up, Catholic and Protestant. The various denominations of Protestant religion were aggregated into Protestant and individuals from other religions and with no religion were excluded from the donation analyses.

Donations Boxes

Figure S1. Donation boxes: Save the Children (left) and Catholic Primary school (right).

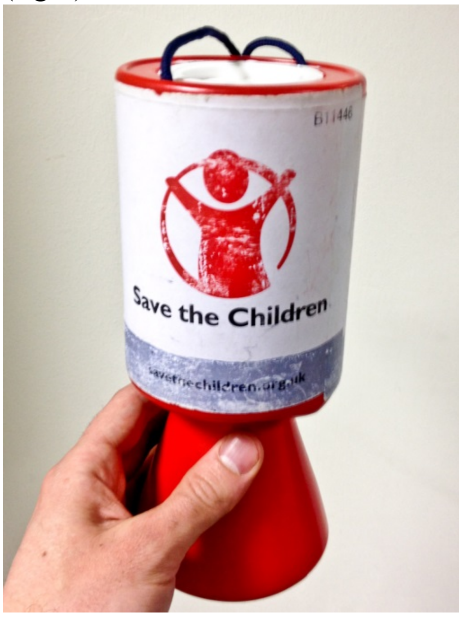

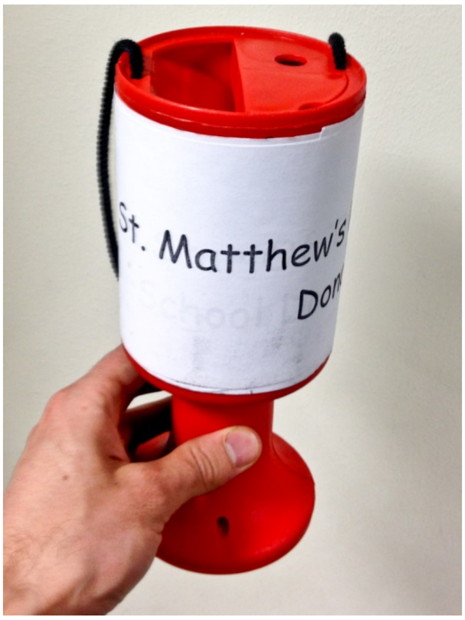

Donations Protocol

Knock twice on the front door and wait for 20 seconds. If no one opens the door proceed to the next house. If someone opens the door say:

Hi, I am doing a student project on neighbourhood well-being in Belfast and it's a questionnaire that should take no more than 10 minutes and for your time we are giving out £5 for answering it.

Wait for reply and if positive add:

At the end you can give some of the money to a local school/charity, but it's up to you and you can decide that in the end.

Start the questionnaire and in the end say:

Ok, that's it. All done. There's just a consent form in the end for you to sign if you consent for this to be used in research and where it also explains that all the data collected is anonymous. Thank you very much and here's the money

Hand them the 5 pound coins, bring the donation box forward and say:

Today we are collecting money for SCHOOL NAME / SAVE THE CHILDREN. Would you like to donate some money? It's completely up to you.
